# The Effect of 3000 K LED Lamps on the Photosynthesis and Morphology of Deciduous Tree Species

**DOI:** 10.1002/pei3.70032

**Published:** 2025-02-18

**Authors:** Flóra Kolman, Zoltán Kolláth, Péter Molnár, Anna Skribanek

**Affiliations:** ^1^ ELTE Eötvös Loránd University Budapest Hungary; ^2^ Department of Physics Károly Eszterházy Catholic University Eger Hungary

**Keywords:** artificial light, LED lamp, light pollution, photosynthesis, urban trees

## Abstract

The effect of artificial light at night (ALAN) on plants is a less explored area within light pollution research. This is especially true for the physiological parameters of photosynthesis of woody plants. The physiological and morphological values of nineteen deciduous urban tree species illuminated by street lamps with a color temperature of 3000 K were examined for light‐polluted and non‐light‐polluted leaves. The morphological studies covered leaf macromorphology (leaf length, leaf width, and biomass production) and histological development (height of the dorsal epidermis and palisade parenchyma, width of photosynthesizing ground tissue and the leaf). The fluorescence yield of the photochemical system II and the net photosynthesis and transpiration of the leaves exposed to different light conditions were determined in the photosynthetic physiology studies. The species included in the research react differently to artificial light, some are able to utilize the extra lighting at night, while others are negatively affected. In this way, the species can be grouped according to their sensitivity to light pollution. The impact of street lights on vegetation can be easily detected by the combined treatment of micromorphological and photosynthetic physiology tests, macromorphological values are not suitable parameters.

## 1 Introduction

Urban artificial light at night (ALAN) has been a growing global anthropogenic environmental factor for decades (Kiyofuji and Saitoh 2004; Falchi et al. 2016; Zaimenko et al. 2023). Analyzing its impact is challenging, as the rapid pace of technological development in lighting has been accompanied by the emergence of newer and newer developments in a relatively short time. In addition, artificial lighting at night is not locally uniform, and its extent depends on the intensity, color composition, and position of the luminaires (Cathey and Campbell 1975; Chaney 2002; Briggs 2006; Giavi et al. 2020; Heinen 2021). Since the middle of the last century, high‐pressure sodium lamps have been used for street lighting, nowadays they have been eclipsed by the rise of LEDs (Chaney 2002; Peña‐García and Sędziwy 2017). What is clear about ALAN, however, is that it has disrupted natural light cycles (Bennie et al. 2016). Plants respond to perceived biotic and abiotic environmental factors with biochemical responses. This trait is an essential response to adapt to the environment (Ballaré and Austin 2019). Light and temperature sensing in plants are closely related (Franklin 2009; Legris et al. 2017). Light affects the development of plant parts and the physiological and biochemical properties that occur in plants. Light‐induced morphological changes (leaf thickness, leaf biomass) and biochemical processes interact, but photosynthesis as a cellular process shows less correlation with these processes (Poorter et al. 2019).

Although the intensity of artificial light differs by a significant order of magnitude from that of sunlight, it is still useful for vegetation because it has the wavelength ranges that induce physiological processes. At the right intensity, blue and red wavelengths can affect photosynthetic processes, while far‐red wavelengths can affect photoperiodic processes (Briggs 2006). The effect of artificial light sources depends on the power of the luminaire and the distance. Light intensity decreases significantly with distance from the luminaire (Heinen 2021). ALAN affects the micromorphological development and photosynthetic pigments in leaves of trees in urban environments (Giavi et al. 2020; Zaimenko et al. 2023). Excess light at night increases the growth rate of plants, and its effect is closely related to soil moisture. In areas with lower soil moisture exposed to ALAN, more intensive plant growth is observed (Hey et al. 2020). The presence of artificial light activates plant photoreceptors, thereby altering plant physiology and phenology (Bennie et al. 2016). The relationship between phenological changes and street lights is addressed in several literatures. They all have in common that they observe late discoloration and defoliation of illuminated tree branches (Matzke 1936; Schroeder 1945; Briggs 2006; Raven and Cockell 2006; Škvareninová et al. 2017; Massetti 2018; Meng et al. 2022). The extension of the vegetative stage length is a threat to illuminated plant parts due to frost damage (Chaney 2002; Raven and Cockell 2006). Several studies have also detected early bud break and flowering on illuminated sides of trees (Matzke 1936; Bennie et al. 2018; Meng et al. 2022). Cathey and Campbell observed differential growth responses induced by HPS lamps in 1975. Different illumination areas affect phenological shifts in deciduous tree species to different extents (Lian et al. 2021). These plant responses are observed in the temperate zone, but not in cold and warm areas (Zheng, Chen, and Koh 2021). Global climate change effects and ALAN‐induced shifts in plant growing season amplify each other (Ettinger et al. 2021).

The relationship between nocturnal illumination and plant physiology is one of the less researched areas. Low (3–7 PAR) light levels are sufficient for photosynthesis (Kim et al. 2015). At night, carbon fixation is initiated in leaves close to the light sources (Raven and Cockell 2006; Gaston et al. 2013, 2014). Meravi and Prajapati (2018) observed a decrease in photosynthetic efficiency of tree species under continuous illumination. Research with meadow‐ grass has found that night illumination reduces the efficiency of the plant's photochemical system II and stomatal conductance, while increasing photosynthetic yield and cellular respiration in the dark (Crump et al. 2021). Artificial light exposure to plants negatively affects secondary metabolic processes and photosynthetic electron transport efficiency, affecting chlorophyll and carotenoid levels. (Giavi et al. 2020; Lazzarin et al. 2021). However, these plant responses are species‐, wavelength‐, and light intensity‐dependent (Giavi et al. 2020). Studies with cultivated plants have shown that high artificial light intensity at night leads to lower chlorophyll accumulation in some species and lower electron transport and quantum efficiency during the day than under natural light conditions (Kim et al. 2015), but plants grown at night under low light intensity show the opposite response (higher daytime carbon fixation and more intense stomatal movements compared to control plants) (Kim and Kim 2016).

In addition to individual‐level changes, ALAN also affects ecological phenomena. Studies in herbaceous plants have found that increasing light intensity affects biomass production, while in other species it does not (Sanders et al. 2018; Crump et al. 2021; Speißer, Liu, and van Kleunen 2021; Liu et al. 2022). This is particularly true for invasive spreading plants, which can use the extra light at night as an advantage to spread (Murphy et al. 2022; Abonyo and Oduor 2023). Plant responses induced by night‐time lighting can transform both food webs and plant‐microbe interactions (Sanders et al. 2015; Kwak et al. 2017; Carvalho and Castillo 2018; Li et al. 2023; Wang et al. 2024). Thicker leaves due to additional light at night can increase the density of herbivorous insects in some species (Cieraad et al. 2023). Changes in microbial interactions can affect plant nutrient uptake and resistance, and plant pest reproduction (Sanders et al. 2015; Li et al. 2023; Wang et al. 2024). This means that the species composition of ecosystems can be significantly altered by ALAN (Speißer, Liu, and van Kleunen 2021; Liu et al. 2022).

Research on the effects of artificial light on vegetation at night has mostly been carried out in greenhouse plantations or as controlled field experiments. Real, applied research in the field is scarce (Heinen 2021; Bennie et al. 2016, 2018). When mapping the relationship between plants and ALAN, the problem is to determine the control treatment (Heinen 2021). We analyzed the response of 19 deciduous tree species to ALAN in terms of morphological and photosynthetic parameters of leaves illuminated at different light intensities. Our hypothesis was that more intense illumination at night affects the macromorphological and histological properties of leaves, influencing the structure of the photosynthetic apparatus and the efficiency of its electron transport. It was hypothesized that the main change in leaf photosynthesis is due to light pollution and that the species studied do not respond uniformly to ALAN at the local level, thus allowing species to be grouped according to their sensitivity to night illumination.

## 2 Method

### 2.1 Sample Collection

Our research was carried out in the city centre of Szombathely, Hungary. A total of 19 deciduous tree leaves were collected between 2019 and 2023. Leaves were collected from branches 4.5 m below street lights and from the opposite side (control) of the trees, which receive much less artificial light at night. The reason for sampling from the same tree was to investigate the local effects of ALAN in the light intensity reaching the leaves and to exclude other abiotic ecological factors that could affect the leaf properties under study. The leaves were placed in a dark bag, away from light and in a humid environment for analysis and transported to the laboratory. In all cases, 15–30 min elapsed between sample collection and analysis. The tree species tested and the number of individuals per species tested are listed in Table 1. The number of species included in the study was influenced by the fact that we tried to take into account the skyline and the shading effect of buildings and other trees when collecting leaves. In addition, several species are not specifically classified as street trees and are therefore found in lower numbers in the municipality.

**TABLE 1:** Number of specimens and taxonomic classification of the investigated plant species.

Species	Number of individuals tested	Systematic classification
Elder ( *Sambucus nigra* L.)	2	Dipsacales
Judas tree ( *Cercis siliquastrum* L.)	2	Fabales
Black locust ( *Robinia pseudoacacia* L.)	2	Fabales
Japanese pagoda tree ( *Styphnolobium japonicum* (L.) Schott.)	2	Fabales
Sessile oak ( *Quercus petraea* (Mattuschka) Lieblein)	2	Fagales
Common alder ( *Alnus glutinosa* (L.) Gaertn.)	1	Fagales
Silver birch ( *Betula pendula* Roth)	1	Fagales
Empress tree ( *Paulownia tomentosa* Thunb.)	2	Lamiales
Flowering ash ( *Fraxinus ornus* L)	2	Lamiales
Bigleaf linden ( *Tilia platyphyllos* Scop.)	6	Malvales
Small‐leaved linden ( *Tilia cordata* Mill.)	15	Malvales
Common linden (*Tilia* × *europea* L.)	12	Malvales
American plane tree ( *Platanus hybrida* Brot.)	2	Proteales
Common hackberry ( *Celtis occidentalis* L.)	12	Rosales
Purple‐leaf plum tree ( *Prunus cerasifera* atropurpurea Ehrh.)	3	Rosales
Japanese cherry ( *Prunus serrulata* Lindl.)	2	Rosales
Tree‐of‐heaven ( *Ailanthus altissima* (Mill.) Swingle)	6	Sapindales
Field maple ( *Acer campestre* L.)	2	Sapindales
Horse chestnut ( *Aesculus hippocastanum* L.)	3	Sapindales

### 2.2 Morphological Studies

**FIGURE 1:**
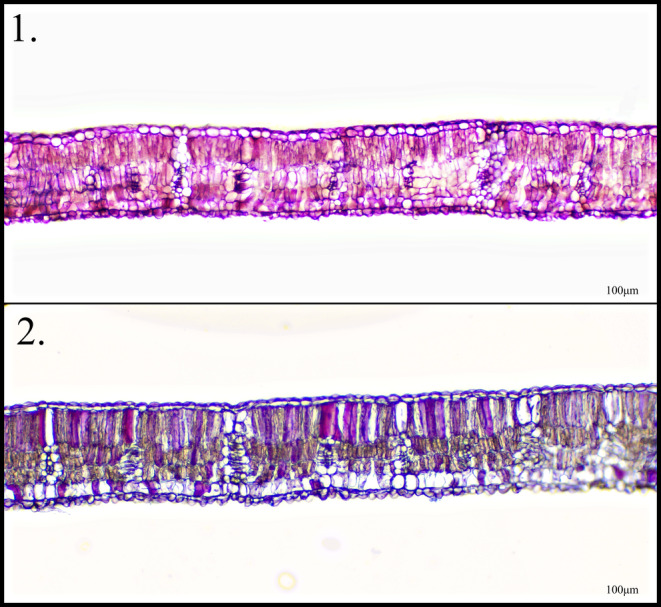
Histological section of black locust ( *Robinia pseudoacacia* L.) after staining. (1) leaf from under the lamp, (2) leaf less exposed to light.

Length, width and weight data were recorded for 10–10 under‐light and control leaves of each species per individual. For micromorphological studies, histological sections of the leaves were prepared and fixed in Canada balsam after Erlich hematoxylin staining (Figure 1). Following staining, the height of the adaxial epidermis and the palisade parenchyma of the leaves, as well as the total photosynthetic ground tissue and leaf thickness, were measured using an Olympus CX33 microscope equipped with an Olympus DP74 camera.

### 2.3 Photosynthetic Studies

The net photosynthesis and transpiration of leaves were measured using a LICOR‐6400 Photosynthesis System. Ten leaves of the individuals were taken from under a street light and ten leaves from the opposite side. Prior to measurements, leaves were adapted in bright light for 2 min. Data were recorded every 20 s with 2‐min measurement periods. Photochemical system II deployment and efficiency were measured using a pulsed modulated fluorescence induction device (imaging PAM, Heinz Walz GmbH, Germany) after dark adaptation of leaves for 20 min. Measurements were performed using blue light illumination of 40 μmol m^−2^ s^−1^ (PAR) every 20 s for 15 periods. The values of F_o_ (fluorescence after dark adaptation); F (fluorescence); F_m_ (maximum fluorescence after dark adaptation) and F_m_′ (maximum fluorescence) were determined. From the obtained results, the maximum quantum efficiency (F_v/_fm), fluorescence yield (Y(II)), photochemical quenching (qp) and non‐photochemical quenching (NPQ) of the photochemical system II were calculated (Table 2). The electron transport efficiency (ETR) of leaves was measured at increasing light intensities of 0–725 μmol m^−2^ s^−1^ (PAR) every 20 s for 12 periods.

**TABLE 2:** Calculated parameters for the deployment and operation of photochemical system II.

Calculated parameters	Formula
Fv/fm (Maximum quantum efficiency of photochemical system II)	(Fm − Fo)/Fm Björkman and Demmig (1987)
Y(II) (fluorescence yield)	(Fm′ − F)/Fm′ Genty, Briantais, and Baker (1989)
qp (photochemical quenching)	(Fm′ − F)/(Fm′ − F0′) Schreiber, Schliwa, and Bilger (1986)
NPQ (non‐photochemical quenching)	(Fm − Fm′)/Fm′ Bilger and Björkman (1991)

Abbreviations: F: fluorescence, F_0_: fluorescence after dark adaptation, F_m_: maximum fluorescence after dark adaptation, F_m_′: maximum fluorescence.

### 2.4 Investigation of the Spectral Composition of Street Lights

**FIGURE 2:**
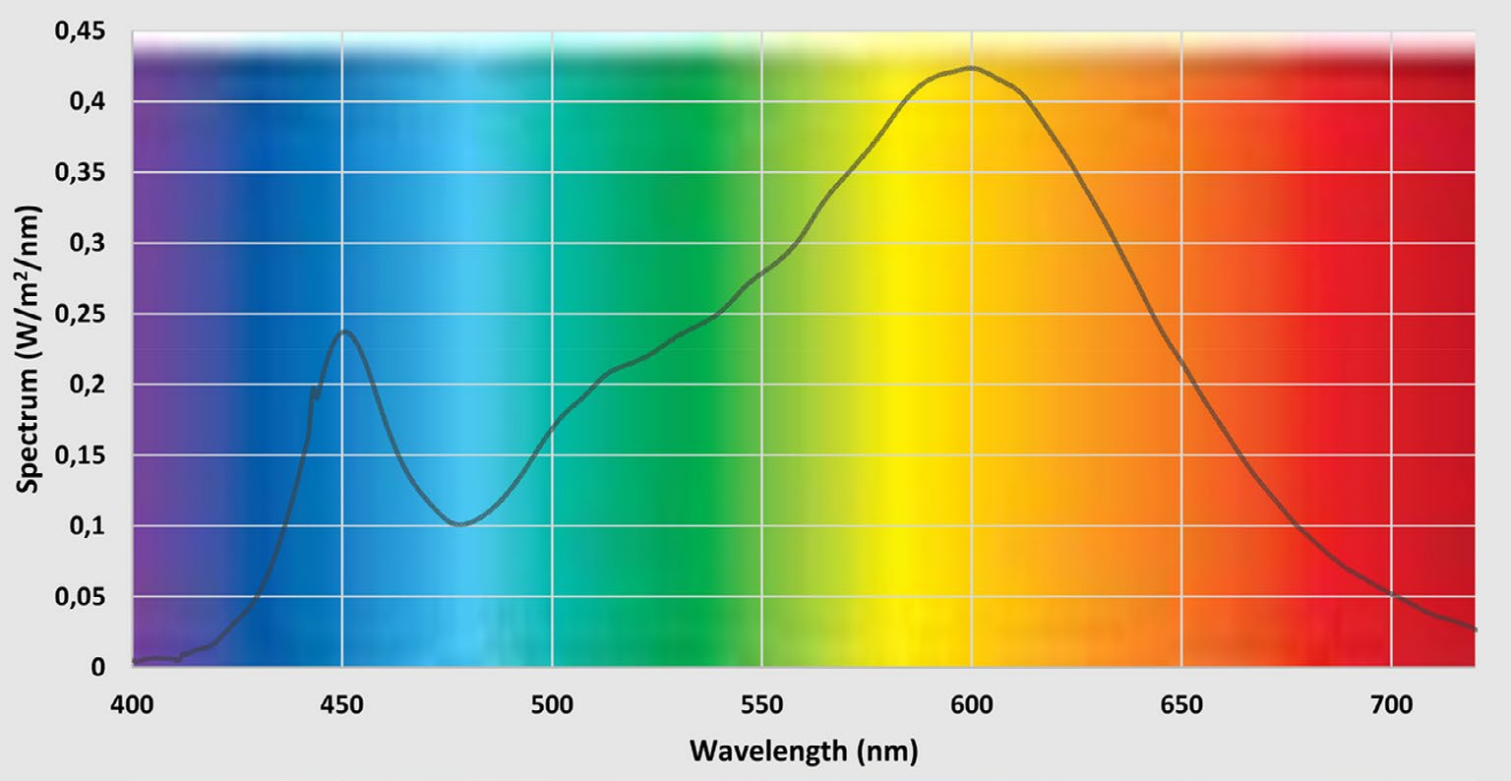
Spectral composition of a 3000 K street LED lamp as a function of the intensity of each wavelength.

Street lighting in the municipality is provided by LED lamps with a color temperature of 3000 K. The spectral composition and photosynthetically active radiation (PAR) of the luminaires were determined using a Konica‐Minolta CS‐2000A Spectroradiometer (Konica Minolta Sensing Inc.) at the height of sampling (Figure 2). The photosynthetically active radiation of the leaves under the lamp was 2.9 PAR, and 0.04 PAR on the opposite side. The measured data were evaluated with Data Management Software CS‐S10w Professional.

### 2.5 Statistical Analysis

Statistical analysis of the morphological and photosynthetic data was performed using PAST 4.03 software. Values of leaves from different illumination environments belonging to the same species were tested for normal distribution at *p* < 0.05 significance level (Kolmogorov–Smirnov and Shapiro–Wilk test). Since the data showed a normal distribution, the measured parameters were subjected to a two‐way ANOVA. First, the effect of artificial light was analyzed for all species, with light as fixed variable, and then the effect of ALAN was analyzed for all species (fixed variable light and species, random to individual). Subsequently, post hoc analysis was used to look for differences between leaf values from the two lighting environments (Tukey test). For Y(II), qp, NPQ, and ETR, data from the last measurement period were examined. The same responses were found when comparing individuals of different species. A difference was detected for one individual. In checking this, it was found that there was an error in the fluorescence measurements and therefore this one individual was excluded from further analysis.

The strength of the effect of night light on leaf morphology and physiology per species was calculated using the formula (*LP*− $\bar{C}$)/σLP, where LP is the value for leaves under the lamp and *C* is the value for leaves on the opposite side. The aim was thus to observe the parameters most affected by light pollution.

**FIGURE 3:**
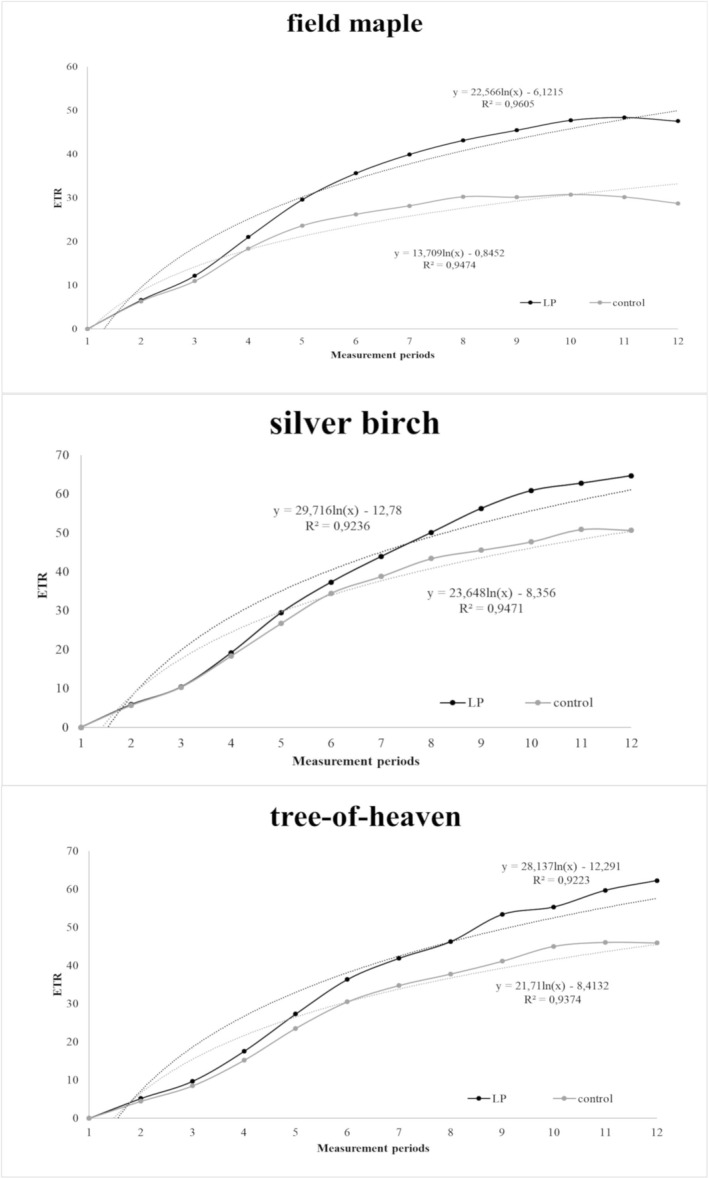
Electron transport rate of photochemical system II in species for which a significant difference between the two light conditions was detected.

For correlations between test parameters, differences in morphological and physiological values of leaves directly illuminated at night and control leaves were subjected to cluster analysis (UPGMA algorithm, Correlation index). For Y(II), qp, and NPQ, differences were also calculated from the values of the last measurement period, ETR values were plotted on graphs, curves were fitted with a straight line, and the difference of the equations of the straight lines was used (Figure 3). The results of the analysis showed a positive correlation between net photosynthesis, Y(II), qp, and ETR values. Species were grouped according to their sensitivity to artificial light using these last four parameters, using cluster analysis (UPGMA algorithm, Euclidean index). Based on the results of the cluster analysis, species were grouped into four categories and a correlation between the irradiance of their place of origin and their sensitivity to light pollution was sought using a Chi‐square test (independent variable: irradiance; dependent variable: category).

## 3 Results

### 3.1 Macromorphology

Differences in leaf length, width and leaf weight between night‐lit and control leaves were detectable in a small proportion of species. Leaves of silver birch under lights were significantly longer, and leaves of horse chestnut receiving additional light at night were significantly longer and wider compared to control leaves. In addition, greater leaf mass was detected in the leaves of horse chestnut under the lamp (Table 3).

**TABLE 3:** Morphological parameters of the studied species.

Tree species	Leaf length (cm)	Leaf width (cm)	Leaf mass (g)	Adaxial epidermis height (μm)	Palisade parenchyma height (μm)	Photosynthetic parenchyma width (μm)	Leaf thickness (μm)
American plane tree_LP	16.92 ± 2.96 *n* = 20	19.64 ± 4.68 *n* = 20	4.78 ± 4.63 n = 20	13.65 ± 2.83*** *n* = 86 *p* = 0.00	77.27 ± 8.45 *n* = 93	159.88 ± 23.18 *n* = 61	211.47 ± 17.54 *n* = 61
American plane tree_control	16.92 ± 3.27 *n* = 20	20.35 ± 5.89 *n* = 20	4.95 ± 2.35 *n* = 20	10.26 ± 2.21 *n* = 131	75.53 ± 9.84 *n* = 126	164.91 ± 23.59 *n* = 74	213.10 ± 24.68 *n* = 76
Bigleaf linden_LP	7.95 ± 0.82 *n* = 60	6.41 ± 0.74 *n* = 60	0.56 ± 0.12 *n* = 60	9.60 ± 1.42 *n* = 60	44.90 ± 4.77*** *n* = 60 *p* = 0.00	131.77 ± 7.78 *n* = 60	164.35 ± 10.39 *n* = 60
Bigleaf linden_control	7.18 ± 0.66 *n* = 60	5.84 ± 0.64 *n* = 60	0.53 ± 0.11 *n* = 60	7.41 ± 1.50 *n* = 60	37.83 ± 3.49 *n* = 60	130.89 ± 9.13 *n* = 60	162.98 ± 8.74 *n* = 60
Black locust_LP	3.77 ± 0.23 *n* = 20	2.33 ± 0.27 *n* = 20	0.14 ± 0.02 *n* = 20	7.67 ± 1.90 *n* = 35	47.71 ± 6.37 *n* = 34	143.39 ± 20.00 *n* = 31	177.70 ± 24.95 *n* = 32
Black locust_control	3.9 ± 0.25 *n* = 20	2.26 ± 0.27 *n* = 20	0.12 ± 0.02 *n* = 20	6.38 ± 1.60 *n* = 33	43.02 ± 4.85 *n* = 40	125.06 ± 9.27 *n* = 34	154.21 ± 8.0 *n* = 33
Common alder_LP	7.57 ± 0.61 *n* = 10	7.08 ± 0.34 *n* = 10	0.78 ± 0.10 *n* = 10	8.99 ± 1.40 *n* = 42	29.33 ± 4.88 *n* = 47	93.77 ± 17.28 *n* = 28	123.58 ± 10.23 *n* = 28
Common alder_control	7.64 ± 2.30 *n* = 10	7.08 ± 1.45 *n* = 10	0.74 ± 0.24 *n* = 10	9.06 ± 1.70 *n* = 52	28.01 ± 2.93 *n* = 49	105.97 ± 7.24 *n* = 28	135.02 ± 7.46 *n* = 28
Common hackberry_LP	7.04 ± 1.49 *n* = 120	3.63 ± 0.80 *n* = 120	0.31 ± 0.12 *n* = 120	22.18 ± 6.03 *n* = 44	71.92 ± 14.25 *n* = 47	98.62 ± 12.34 *n* = 45	191.64 ± 20.97 *n* = 46
Common hackberry_control	7.28 ± 1.35 *n* = 120	3.82 ± 0.73 *n* = 120	0.34 ± 0.12 *n* = 120	23.97 ± 5.98 *n* = 47	67.52 ± 8.69 *n* = 48	102.59 ± 8.85 *n* = 45	193.79 ± 9.21 *n* = 47
Common linden_LP	7.42 ± 0.82 *n* = 120	5.91 ± 0.63 *n* = 120	0.52 ± 1.11 *n* = 120	16.38 ± 3.07 *n* = 55	43.61 ± 5.88 *n* = 54	106.21 ± 14.13 *n* = 54	139.92 ± 15.01 *n* = 54
Common linden_control	6.76 ± 1.03 *n* = 120	5.55 ± 0.73 *n* = 120	0.47 ± 0.15 *n* = 120	13.78 ± 2.01 *n* = 83	37.81 ± 4.40 *n* = 93	116.97 ± 94.47 *n* = 90	141.54 ± 10.61 *n* = 87
Elder_LP	16.83 ± 2.25 *n* = 20	13.71 ± 1.99 *n* = 20	20.03 ± 0.29 *n* = 20	15.73 ± 2.55*** *n* = 46 *p* = 0.00	33.68 ± 3.84* *n* = 47 *p* = 0.02	145.22 ± 21.59 *n* = 27	189.17 ± 23.29 *n* = 27
Elder_control	19.11 ± 1.89 *n* = 20	15.24 ± 2.40 *n* = 20	2.34 ± 0.52 *n* = 20	9.87 ± 1.86 *n* = 47	27.24 ± 3.04 *n* = 53	125.89 ± 29.71 *n* = 27	167.38 ± 16.14 *n* = 27
Empress tree_LP	16.15 ± 1.58 *n* = 20	11.6 ± 1.62 *n* = 20	2.34 ± 0.81 *n* = 20	10.83 ± 2.33 *n* = 80	53.47 ± 9.40 *n* = 69	123.71 ± 18.67 *n* = 58	156.51 ± 22.18 *n* = 58
Empress tree_control	14.37 ± 1.86 *n* = 20	10.55 ± 1.51 *n* = 20	1.87 ± 0.65 *n* = 20	8.35 ± 1.91 *n* = 69	59.59 ± 6.40** *n* = 61 *p* = 0.003	146.78 ± 21.96 *n* = 44	180.85 ± 26.33 *n* = 44
Field maple_LP	15.47 ± 1.90 *n* = 20	20.49 ± 2.99* *n* = 20 *p* = 0.02	2.88 ± 0.60 *n* = 20	10.07 ± 1.48 *n* = 37	31.72 ± 5.73 *n* = 39	87.74 ± 11.54 *n* = 39	118.23 ± 12.86 *n* = 39
Field maple_control	14.66 ± 0.81 *n* = 20	17.93 ± 1.50 *n* = 20	2.33 ± 6.50 *n* = 20	9.92 ± 1.48 *n* = 42	32.60 ± 3.53 *n* = 43	89.19 ± 5.94 *n* = 42	122.96 ± 7.34 *n* = 42
Flowering ash_LP	9.88 ± 0.84 *n* = 20	4.17 ± 0.52 *n* = 20	3.49 ± 0.64 *n* = 20	11.02 ± 2.08 *n* = 37	65.19 ± 11.57 *n* = 38	170.16 ± 18.28 *n* = 36	208.82 ± 19.37 *n* = 36
Flowering ash_control	10.53 ± 0.48 *n* = 20	3.29 ± 0.33 *n* = 20	4.17 ± 2.35 *n* = 20	8.06 ± 1.72 *n* = 38	49.52 ± 5.00 *n* = 40	145.03 ± 15.25 *n* = 40	179.37 ± 18.37 *n* = 37
Horse chestnut_LP	36 ± 4.69*** *n* = 20 *p* = 0.00	23.55 ± 2.75*** *n* = 20 *p* = 0.00	14.69 ± 3.80*** *n* = 20 *p* = 0.00	11.48 ± 2.71 *n* = 48	50.39 ± 6.76** *n* = 38 *p* = 0.003	137.78 ± 26.27 *n* = 24	173.28 ± 25.49 *n* = 24
Horse chestnut_control	29.05 ± 5.26 *n* = 20	19.46 ± 3.15 *n* = 20	11.34 ± 3.15 *n* = 20	10.46 ± 3.40 *n* = 44	42.51 ± 5.60 *n* = 41	137.31 ± 13.43 *n* = 21	175.33 ± 16.03 *n* = 21
Japanese cherry_LP	13.07 ± 1.43 *n* = 20	6.17 ± 0.38 *n* = 20	1.48 ± 0.24 *n* = 20	18.89 ± 4.45 *n* = 76	53.28 ± 6.37** *n* = 84 *p* = 0.01	174.08 ± 15.19 *n* = 59	230.24 ± 17.08 *n* = 58
Japanese cherry_control	13.25 ± 2.18 *n* = 20	6.31 ± 0.84 *n* = 20	1.48 ± 0.49 *n* = 20	17.83 ± 2.15 *n* = 76	47.57 ± 4.92 *n* = 78	174.54 ± 24.23 *n* = 63	227.64 ± 21.36 *n* = 63
Japanese pagoda tree_LP	5.6 ± 0.57 *n* = 20	2.59 ± 0.21 *n* = 20	0.18 ± 0.04 *n* = 20	11.91 ± 1.53*** *n* = 78 *p* = 0.00	25.59 ± 2.46*** *n* = 84 *p* = 0.00	123.4 ± 15.84 *n* = 56	162.42 ± 16.45 *n* = 55
Japanese pagoda tree_control	5.31 ± 0.66 *n* = 20	2.23 ± 0.25 *n* = 20	0.16 ± 0.03 *n* = 20	8.17 ± 1.13 *n* = 74	20.00 ± 2.14 *n* = 94	122.81 ± 12.45 *n* = 56	157.70 ± 13.82 *n* = 52
Judas tree_LP	7.65 ± 1.13 *n* = 20	8.31 ± 1.05 *n* = 20	1.07 ± 0.29 *n* = 20	12.80 ± 2.33 *n* = 104	48.37 ± 4.91*** *n* = 97 *p* = 0.00	132.16 ± 14.64 *n* = 68	170.45 ± 14.89 *n* = 68
Judas tree_control	8.11 ± 1.78 *n* = 20	8.42 ± 1.38 *n* = 20	1.17 ± 0.46 *n* = 20	11.63 ± 2.59 *n* = 95	42.65 ± 5.40 *n* = 102	141.94 ± 15.12 *n* = 65	180.48 ± 13.88 *n* = 66
Purple‐leaf plum tree_LP	6.23 ± 0.84 *n* = 30	3.66 ± 0.40 *n* = 30	0.26 ± 0.05 *n* = 30	12.90 ± 2.09 *n* = 93	34.64 ± 4.31 *n* = 102	96.55 ± 15.16 *n* = 66	136.71 ± 19.02 *n* = 68
Purple‐leaf plum tree_control	6.68 ± 0.60 *n* = 30	3.72 ± 0.32 *n* = 30	0.27 ± 0.06 *n* = 30	12.35 ± 1.84 *n* = 66	34.05 ± 5.13 *n* = 77	86.22 ± 12.33 *n* = 46	120.70 ± 15.62 *n* = 49
Sessile oak_LP	20.88 ± 1.98 *n* = 20	14.23 ± 2.21 *n* = 20	2.30 ± 0.51 *n* = 20	21.71 ± 2.76*** *n* = 45 *p* = 0.00	35.56 ± 6.07 *n* = 45	100.54 ± 9.80 *n* = 45	149.30 ± 10.47 *n* = 45
Sessile oak_control	20.04 ± 1.37 *n* = 20	12.66 ± 1.56 *n* = 20	2.03 ± 0.52 *n* = 20	19.18 ± 2.82 *n* = 41	48.231.89 ± * *n* = 43 *p* = 0.04	107.56 ± 14.70 *n* = 42	157.52 ± 17.08 *n* = 40
Silver birch_LP	6.93 ± 0.56 *n* = 10	4.50 ± 0.56 *n* = 10	0.30 ± 0.05 *n* = 10	11.95 ± 1.73 *n* = 49	35.50 ± 3.53*** *n* = 68 *p* = 0.00	128.07 ± 23.59 *n* = 34	168.73 ± 10.87 *n* = 32
Silver birch_control	6.21 ± 0.43 *n* = 10	3.94 ± 0.24 *n* = 10	0.22 ± 0.04 *n* = 10	9.39 ± 1.63 *n* = 1043	28.61 ± 2.58 *n* = 1058	113.71 ± 11.85 *n* = 27	145.80 ± 12.11 *n = 10*29
Small‐leaved linden_LP	6.27 ± 0.70 *n* = 150	5.56 ± 0.60 *n* = 150	0.45 ± 0.09 *n* = 150	11.58 ± 2.17 *n* = 85	44.25 ± 5.12 *n* = 90	144.99 ± 14.23 *n* = 84	185.66 ± 20.24 *n* = 84
Small‐leaved linden_control	6.25 ± 1.02 *n* = 150	5.63 ± 0.75 *n* = 150	0.46 ± 1.22 *n* = 150	11.60 ± 2.16 *n* = 102	49.22 ± 6.10*** *n* = 108 *p* = 0.00	137.71 ± 16.78 *n* = 104	175.61 ± 16.27 *n* = 107
Tree‐of‐heaven_LP	12.43 ± 2.39 *n* = 60	5.35 ± 0.95 *n* = 60	1.09 ± 0.48 *n* = 60	13.46 ± 2.39 *n* = 276	76.26 ± 9.91 *n* = 279	171.45 ± 23.02*** *n* = 271 *p* = 0.00	223.21 ± 25.28*** *n* = 264 *p* = 0.00
Tree‐of‐heaven_control	11.86 ± 2.25 *n* = 60	5.28 ± 0.68 *n* = 60	0.94 ± 0.36 *n* = 60	12.38 ± 2.37 *n* = 205	73.75 ± 14.87 *n* = 198	155.48 ± 22.88 *n* = 192	206.26 ± 27.24 *n* = 192

Abbreviations: control, Leaves less exposed to light; LP, leaves from under the street lamps.

*p < 0.05; **p < 0.01; ***p < 0.001.

### 3.2 Micromorphology

Among the histological parameters, four species (American plane tree, elder, Japanese pagoda tree, sessile oak) showed differences in the adaxial epidermis in response to stronger illumination at night. In these species, adaxial epidermal cells of illuminated leaves were significantly higher compared to control leaves. The height of the palisade parenchyma showed greater variation: in eight species (bigleaf linden, elder, empress tree, horse chestnut, Japanese cherry, Japanese pagoda tree, judas tree, silver birch), cells with higher light intensity at night were taller, while in two species (sessile oak, small‐leaved linden) cells of the control leaves were higher compared to the opposite side. For nine species (American plane tree, black locust, common alder, common hackberry, common linden, filed maple, flowering ash, purple‐leaf‐plum tree, tree‐of‐heaven), no differences were detected in the parameter tests. The thickness of the photosynthetic ground tissue was similar in most species at the two light intensities, with the exception of the tree‐of‐heaven, where the photosynthetic basal tissue of the leaves under the lamp was significantly larger than that of the control leaves. In the analysis of leaf thickness, we also found that, as with photosynthetic base tissue, the only species under the lamp that had a more developed leaf cross‐section was the tree‐of‐heaven. No differences were detected in the other species (Table 3).

### 3.3 Photosynthesis

The maximum quantum efficiency of photochemical system II (Fv/fm) did not differ between directly illuminated and less light‐polluted leaves in most species. The test parameter showed no correlation with the results based on fluorescence and carbon fixation in several species. In the maximum quantum efficiency of different lighting environments, the Japanese pagoda tree, silver birch, and the purple leaf plum tree had a clearly positive response to artificial light at night, while the values of the leaves on the opposite side of the tree to the lamp were significantly higher. The fluorescence yield of highly light opolluted leaves was significantly higher for common linden and elder, significantly lower for American plane tree and black locust compared to leaves on the opposite side. Leaves of three species (field maple, silver birch and tree‐of‐heaven) showed higher values for electron transport efficiency under the lamp. No difference was detected at all for the difference in photochemical system II for bigleaf linden, common alder, empress tree, flowering ash, horse chestnut, Japanese cherry, judas tree, sessile oak (Table 4).

**TABLE 4:** The construction and efficiency of the photochemical system II, as well as the rate of transpiration in the examined species in two different lighting environments.

Tree species	Fv/fm (rel.)	qp (highest value)	Y(II) (highest value)	ETR (highest value)	NPQ (highest value)	Transpiration (mikromol H_2_Os^−1^ m^−2^)
American plane tree_LP	0.791 ± 0.01 *n* = 60	0.886 ± 0.13 *n* = 55	0.601 ± 0.03 *n* = 60	41.69 ± 9.85 *n* = 60	0.713 ± 0.17*** *n* = 60 ***p*** **=** **0** **.** **0** **0**	0.003 ± 0.001 *n* = 187
American plane tree_control	0.795 ± 0.01 *n* = 30	0.938 ± 0.04*** *n* = 55 ***p*** **=** **0.** **0** **0**	0.608 ± 0.03*** *n* = 60 ***p*** **=** **0.00**	48.71 ± 9.46 *n* = 60	0.694 ± 0.19 *n* = 60	0.003 ± 0.001 *n* = 187
Bigleaf linden_LP	0,788 ± 0.01 *n* = 21	0.906 ± 0.05 *n* = 216	0.547 ± 0.10 *n* = 216	44.39 ± 4.96 *n* = 216	0.749 ± 0.18 *n* = 216	0.194 ± 0.18 *n* = 231
Bigleaf linden_control	0.778 ± 0.02 *n* = 216	0.920 ± 0.03 *n* = 216	0.562 ± 0.04 *n* = 216	44.55 ± 9.15 *n* = 216	0.706 ± 0.21 *n* = 216	0.164 ± 0.08 *n* = 231
Black locust_LP	0.769 ± 0.28 *n* = 120	0.964 ± 0.01	0.596 ± 0.04 *n* = 120	52.36 ± 15.07 *n* = 120	0.416 ± 0.11 *n* = 120	0.65 ± 0.41 *n* = 228
Black locust_control	0.784 ± 0.13 *n* = 120	0.965 ± 0.01 *n* = 120	0.614 ± 0.03*** *n* = 120 ***p*** **=** **0.00**	53.83 ± 13.00 *n* = 120	0.411 ± 0.09 *n* = 120	0.60 ± 0.24 *n* = 228
Common alder_LP	0.801 ± 0.01 *n* = 30	0.925 ± 0.03 *n* = 30	0.601 ± 0.04 *n* = 30	49.05 ± 7.96 *n* = 30	0.725 ± 0.20 *n* = 30	0.130 ± 0.07 *n* = 121
Common alder_control	0.797 ± 0.01 *n* = 30	0.925±.020 *n* = 30	0.591 ± 0.02 *n* = 30	50.85 ± 7.05 *n* = 30	0.881 ± 0.21 *n* = 30	0.132 ± 0.04 *n* = 121
Common hackberry_LP	0.780 ± 0.02 *n* = 453	0.923 ± 0.04 *n* = 408	0.521 ± 0.08 *n* = 408	103.21 ± 26.26 *n* = 408	0.823 ± 0.23** *n* = 408 ***p*** **=** **0.00**	0.144 ± 0.10 *n* = 1729
Common hackberry_control	0.776 ± 0.03 *n* = 453	0.923 ± 0.01 *n* = 408	0.522 ± 0.08 *n* = 408	111.21 ± 23.03 *n* = 408	0.744 ± 0.21 *n* = 408	0.180 ± 0.14 *n* = 1729
Common linden_LP	0.793 ± 0.03 *n* = 150	0.797 ± 0.11 *n* = 330	0.511 ± 0.06*** *n* = 390 **p** **=** **0.00**	36.15 ± 6.82 *n* = 330	1453 ± 0.49** *n* = 330 ***p*** **=** **0.002**	0.288 ± 0.23 *n* = 1071
Common linden_control	0.787 ± 0.02 *n* = 150	0.837 ± 0.07 *n* = 330	0.505 ± 0.06 *n* = 390	42.26 ± 8.49 *n* = 330	1371 ± 0.36 *n* = 330	0.377 ± 0.21 *n* = 1071
Elder_LP	0.783 ± 0.01 *n* = 60	0.799 ± 0.08* *n* = 60 **p** **=** **0.03**	0.536 ± 0.05** *n* = 60 ***p*** **=** **0.009**	28.20 ± 6.89 *n* = 60	1357 ± 0.36 *n* = 60	0.245 ± 0.07 *n* = 110
Elder_control	0.775 ± 0.01 *n* = 60	0.718 ± 0.09 *n* = 60	0.476 ± 0.04 *n* = 60	21.42 ± 4.15 *n* = 60	1621 ± 0.33 *n* = 60	0.275 ± 0.15 *n* = 110
Empress tree_LP	0.795 ± 0.03 *n* = 60	0.952 ± 0.03 *n* = 60	0.592 ± 0.03 *n* = 60	65.18 ± 8.45 *n* = 60	0.541 ± 0.11 *n* = 60	1839 ± 0.36*** *n* = 121 ***p*** **=** **0.00**
Empress tree_control	0.786 ± 0.03 *n* = 60	0.941 ± 0.03 *n* = 60	0.593 ± 0.03 *n* = 60	59.87 ± 14.53 *n* = 60	0.60 ± 0.11 *n* = 60	1296 ± 0.52 *n* = 121
Field maple_LP	0.798 ± 0.02 *n* = 60	0.947 ± 0.03* *n* = 60 **p** **=** **0.01**	0.602 ± 0.04 *n* = 60	47.57 ± 14.12*** *n* = 60 **p** **= 0.00**	0.629 ± 0.29 *n* = 60	0.421 ± 0.25*** *n* = 110 ***p*** **=** **0.00**
Field maple_control	0.782 ± 0.02 *n* = 60	0.888 ± 0.10 *n* = 60	0.551 ± 0.07 *n* = 60	29.68 ± 10.63 *n* = 60	0.829 ± 0.36 *n* = 60	0.237 ± 0.08 *n* = 110
Flowering ash_LP	0.798 ± 0.1 *n* = 60	0.911 ± 0.05 *n* = 60	0.522 ± 0.05 *n* = 60	36.45 ± 7.26 *n* = 60	0.958 ± 0.36 *n* = 60	0.354 ± 0.38 *n* = 120
Flowering ash_control	0.779 ± 0.02 *n* = 60	0.855 ± 0.11 *n* = 60	0.522 ± 0.07 *n* = 60	35.60 ± 7.80 *n* = 60	1073 ± 0.53 *n* = 60	0.329 ± 0.59 *n* = 120
Horse chestnut_LP	0.762 ± 0.01 *n* = 180	0.893 ± 0.02 *n* = 180	0.550±.0.02 *n* = 180	32.144 ± 7.75 *n* = 180	0.790 ± 0.12 *n* = 180	0.332 ± 0.17 *n* = 131
Horse chestnut_control	0.779 ± 0.02 *n* = 180	0.892 ± 0.04 *n* = 180	0.568 ± 0.05 *n* = 180	41.54 ± 8.67 *n* = 180	0.888 ± 0.22 *n* = 180	0.242 ± 0.09 *n* = 131
Japanese cherry_LP	0.717 ± 0.03 *n* = 60	0.829 ± 0.08 *n* = 60	0.490 ± 0.05 *n* = 60	35.6 ± 3.75 *n* = 60	1214 ± 0.94 *n* = 60	0.440 ± 0.32 *n* = 231
Japanese cherry_control	0.716 ± 0.04 *n* = 60	0.867 ± 0.09 *n* = 60	0.539 ± 0.02 *n* = 60	36.84 ± 2.64 *n* = 60	0.782 ± 0.02 *n* = 60	0.438 ± 0.47 *n* = 231
Japanese pagoda tree_LP	0.783 ± 0.02** *n* = 60 **p** **= 0.009**	0.940 ± 0.05 *n* = 60	0.602 ± 0.03 *n* = 60	48.72 ± 11.17 *n* = 60	0.517 ± 0.13 *n* = 60	0.412 ± 0.90 *n* = 198
Japanese pagoda tree_control	0.760 ± 0.04 *n* = 60	0.937 ± 0.04 *n* = 60	0.532 ± 0.04 *n* = 60	43.73 ± 11.84 *n* = 60	0.492 ± 0.18 *n* = 60	0.317 ± 0.96 *n* = 198
Judas tree_LP	0.792 ± 0.03 *n* = 60	0.934 ± 0.04 *n* = 60	0.563 ± 0.04 *n* = 60	49.45 ± 10.98 *n* = 60	0.677 ± 0.23 *n* = 60	0.508 ± 0.24*** *n* = 220 *p* **= 0.00**
Judas tree_control	0.799 ± 0.02 *n* = 60	0.943 ± 0.02 *n* = 60	0.591 ± 0.04 *n* = 60	49.34 ± 9.14 *n* = 60	0.605 ± 0.17 *n* = 60	0.418 ± 0.18 *n* = 220
Purple‐leaf plum tree_LP	0.749 ± 0.03 *n* = 90	0.839 ± 0.017 *n* = 90	0.538 ± 0.08 *n* = 90	61.74 ± 14.69 *n* = 90	0.822 ± 0.30 *n* = 90	0.136 ± 0.06 *n* = 220
Purple‐leaf plum tree_control	0.773 ± 0.03* *n* = 90 ***p*** **= 0.02**	0.833 ± 0.14 *n* = 60	0.534 ± 0.07 *n* = 90	51.81 ± 13.65 *n* = 90	1066 ± 0.77 *n* = 90	0.137 ± 0.07 *n* = 220
Sessile oak_LP	0.804 ± 0.01 *n* = 60	0.948 ± 0.03 *n* = 60	0.548 ± 0.07 *n* = 60	31.90 ± 9.59 *n* = 60	0.633 ± 0.24 *n* = 60	0.153 ± 0.12 *n* = 109
Sessile oak_co*n*trol	0.793 ± 0.02 *n* = 60	0.946 ± 0.06 *n* = 60	0.595 ± 0.09 *n* = 60	41.79 ± 11.59 *n* = 60	0.561 ± 0.35 *n* = 60	0.160 ± 0.18 *n* = 109
Silver birch_LP	0.750 ± 0.02 *n* = 30	0.896 ± 0.03 *n* = 30	0.583 ± 0.02 *n* = 30	64.65 ± 6.88** *n* = 30 ***p*** **= 0.006**	0.699 ± 0.10 *n* = 30	0.076 ± 0.06 *n* = 110
Silver birch_control	0.782 ± 0.04** *n* = 30 ***p*** **= 0.001**	0.873 ± 0.08 *n* = 30	0.562 ± 0.02 *n* = 30	50.91 ± 9.01 *n* = 30	1003 ± 0.69 *n* = 30	0.153 ± 0.14 *n* = 110
Small‐leaved linden_LP	0.778 ± 0.05 *n* = 191	0.704 ± 0.14 *n* = 191	0.491 ± 0.08 *n* = 191	43.43 ± 8.28 *n* = 191	1236 ± 0.52 *n* = 191	0.157 ± 0.15 *n* = 600
Small‐leaved linden_control	0.779 ± 0.05 *n* = 191	0.921 ± 0.04*** *n* = 191 ***p*** **= 0.00**	0.488 ± 0.09 *n* = 191	44.51 ± 9.63 *n* = 191	1354 ± 0.58 *n* = 191	0.222 ± 0.18 *n* = 600
Tree‐of‐heaven_LP	0.760 ± 0.03 *n* = 180	0.937 ± 0.03 *n* = 180	0.605 ± 0.03 *n* = 180	63.29 ± 14.69*** *n* = 180 **p** **= 0.0004**	0.436 ± 0.12 *n* = 180	0.259 ± 0.18 *n* = 206
Tree‐of‐heaven_control	0.772 ± 0.02 *n* = 180	0.923 ± 0.03 *n* = 180	0.582 ± 0.08 *n* = 180	54.22 ± 13.41 *n* = 180	0.488 ± 0.20 *n* = 180	0.147 ± 0.06 *n* = 206

Abbreviations: control, leaves less exposed to light; LP, leaves from under the street lamps.

*p < 0.05; **p < 0.01; ***p < 0.001.

**FIGURE 4:**
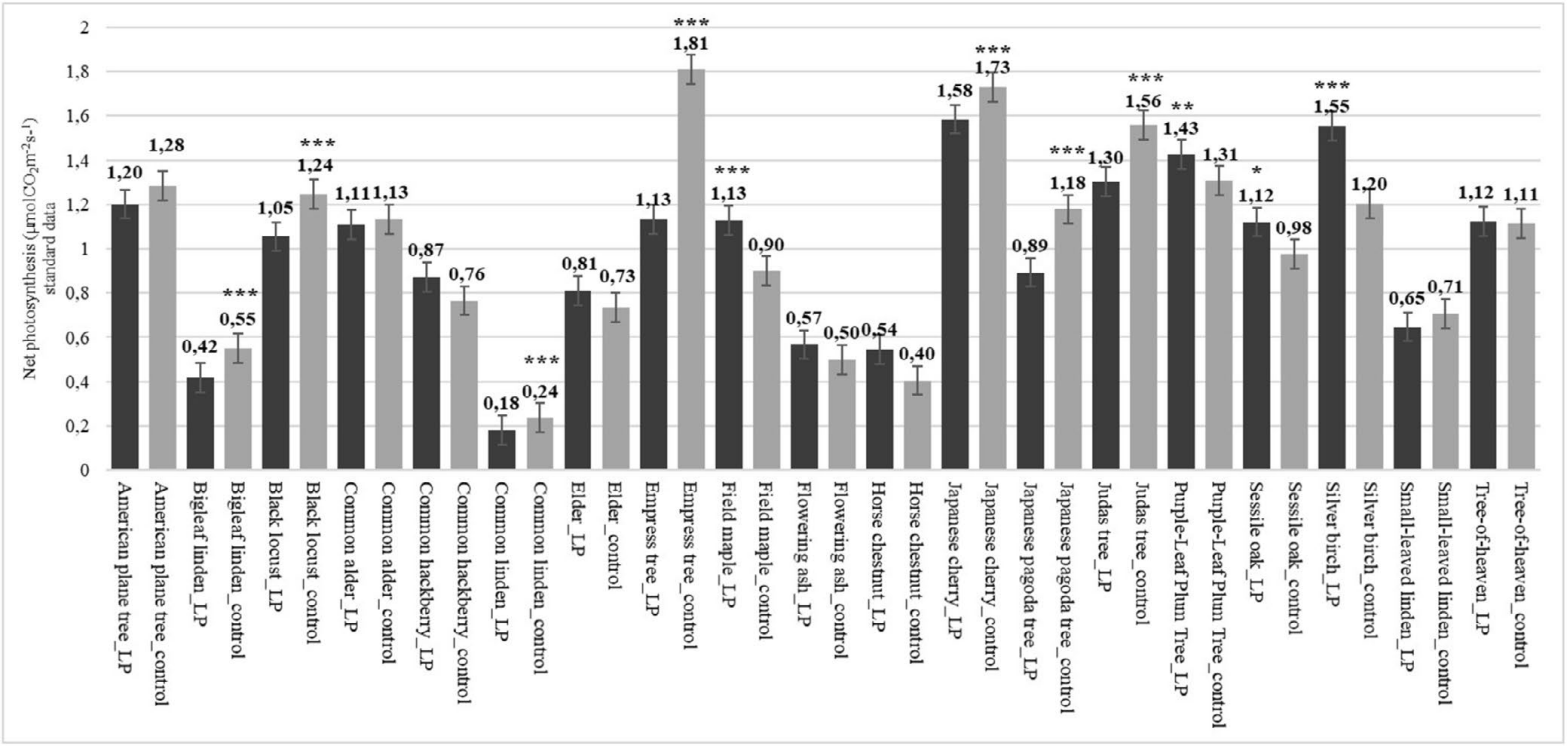
Net photosynthesis of the investigated species in different lighting environments at night. control: leaves less exposed to light; LP: leaves from under the street lamps, *p < 0.05; **p < 0.01; ***p < 0.001).

There were differences in net photosynthetic performance for 11 species. It was found to be higher for highly light‐polluted leaves in four species (filed maple, sessile oak, purple‐leaf plum tree), with bigleaf linden, black locust, common linden, empress tree, Japanese cherry, Japanese pagoda tree, Judas tree having significantly lower photosynthetic yields under lamp conditions compared to less light‐polluted leaves (Figure 4). Transpiration values differed for three species (American plane tree; black locust; common alder; elder; flowering ash; Japanese cherry; purple‐leaf plum tree and sessile oak) when comparing leaves that were strongly and less illuminated at night. In all three cases, the values were significantly higher for leaves under lights. Net photosynthesis and transpiration values were similar in these three species, while no correlation between the two parameters was detected in the other species (Table 4, Figure 4).

### 3.4 Comparison of Test Parameters

**FIGURE 5:**
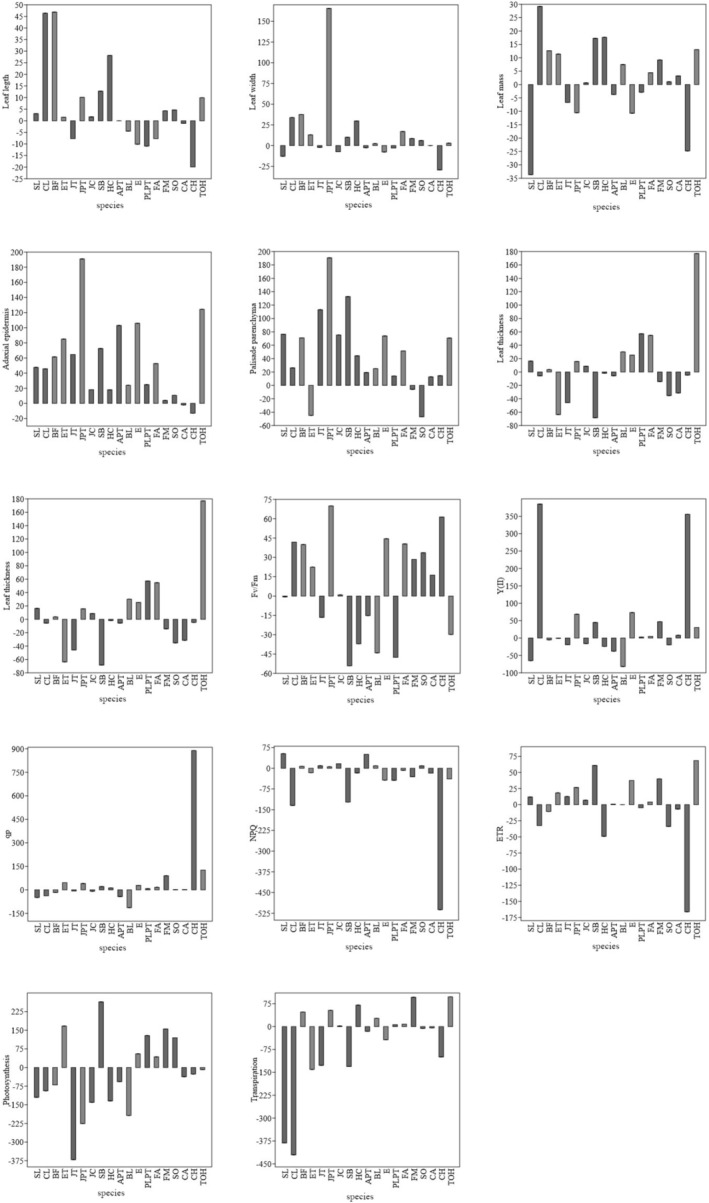
The strength of the effect of test parameters on species. APT: American plane tree, BF: bigleaf linden, BL: black locust, CA: common alder, CH: common hackberry, CL: common linden, E: elder, ET: empress tree, ETR: Rate of electron transport chain, FA: flowering ash, FM: field maple, Fv/fm: Maximum quantum efficiency of photosystem II, HC: horse chestnut, JC: Japanese cherry, JPT: Japanese pagoda tree, JT: juda tree, NPQ: Non‐photochemical quenching, PLPT: purple‐leaf plum tree, qp: Photochemical quenching, SB: silver birch, SL: small‐leaved linden, SO: sessile oak, TOH: tree‐of‐heaven, Y(II): Fluorescence yield.

The strength of the effect of artificial light at night on the test parameters varied between species. Characteristics based on fluorescence values (Y(II), qp, ETR, NPQ) and carbon fixation measurements (net photosynthesis, transpiration) were influenced by the level of irradiation at night in most species, and also by the height of adaxial epidermis cells and palisade parenchyma. The maximum quantum efficiency of photochemical system II (Fv/fm) and other morphological characteristics had little effect (Figure 5).

In correlation analysis between parameters, leaf macromorphology showed no correlation with physiological and micromorphological values. In histological and plant physiological studies, correlations were found between the fluorescence yield of the photochemical systems II (Y(II)), photochemical quenching (qp), the efficiency of the electron transport (ETR) and net photosynthesis. Taking into account the differential values of these latter traits, the responses of tree species to artificial nighttime illumination were mapped by individual using cluster analysis. Individuals of the same species were grouped separately, as they behaved similarly, no outliers were found, and therefore species difference averages are shown.

**FIGURE 6:**
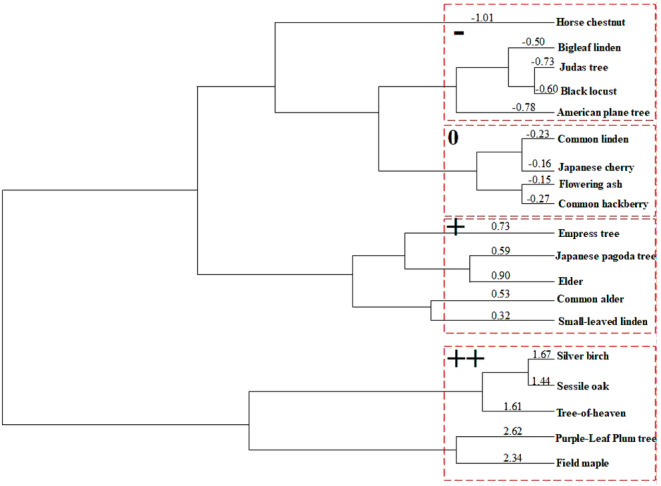
Classification of the studied species into groups based on their sensitivity to light pollution. Numbers represent the average of the difference values of fluorescence yield (Y(II)), photochemical quenching (qp), electron transport efficiency (ETR) and net photosynthetic yield of leaves under the lamp and on the opposite side. −: Negative response to direct lighting at night, +: Weak positive response to direct lighting at night, ++: Strong positive response to direct lighting at night, 0: Neutral response to direct lighting at night.

The results show that species can be divided into four distinct groups based on their response to light pollution. Horse chestnut; bigleaf linden; judas tree; black locust and American plane tree responded negatively to light pollution (mean difference between night‐lit and control leaves ranged from −1.00 to −0.50). Common linden, Japanese cherry, flowering ash and common hackberry were neutrally affected by overnight overexposure (mean difference between night‐lit and control leaves ranged from −0,30 to −0,00). Weak positive responses were observed for empress tree; Japanese pagoda tree; elder, common alder and small‐leaved linden (mean difference between night‐lit and control leaves ranged from −0.30 to −0.90). The silver birch; sessile oak; tree‐of‐heaven; purple‐leaf plum tree and filed maple were able to utilize artificial light at night in their photomorphogenetic processes (mean difference between night‐lit and control leaves was 1.30–2.70) (Figure 6).

The origin and taxonomic classification of the plants did not show any correlation with the response to light pollution (*p* = 0.79 and *p* = 0.34).

## 4 Discussion

Our tests were carried out on the highly and less light‐polluted leaves of 19 deciduous tree species with 3000 K street LED lights. Our aim was to map the local effects of light pollution in the light of different levels of photosynthetically active radiation reaching the leaves at night. During our measurements, morphological and photosynthetic data were recorded. Different plant species have different light requirements, so that even low levels of artificial irradiation can trigger physiological processes in some species (Chaney 2002; Kim et al. 2015; Segrestin et al. 2021).

### 4.1 Morphology

Leaf length and width are independent of the lighting conditions. In the test species, direct illumination at night is not associated with higher biomass production. The values from the efficacy studies also confirm that leaf macromorphology, the size of photosynthetic ground tissue and leaf thickness are less affected by the level of night illumination. This may be explained by the fact that morphological changes in response to ALAN occur at the individual level (Poorter et al. 2019). However, it should be noted that the adaxial epidermis is generally higher in leaves under the lamp, even if no significant difference was detected in the majority of species. Furthermore, the palisade parenchyma size of leaves from the two illumination environments was also larger in leaves under the lamp in the majority of the species studied, supporting that night‐time artificial light affects morphological development, although the development of the latter parameter does not correlate with net photosynthetic yield (Segrestin et al. 2021).

### 4.2 Photosynthetic Physiology

The maximum quantum efficiency of photochemical system II (Fv/fm) and the rate of transpiration of the species are independent of the illumination environment. The latter contradicts the observation of Chaney (2002) that stomatal openness increases in deciduous trees under the influence of artificial illumination at night. Although many literatures consider the Fv/fm value for the photochemical system II deployment, it was not found to be an appropriate indicator in our study. Values for photosystem function (qp; Y(II); ETR) and net photosynthetic yield based on fluorescence assays are affected by artificial light at night, but the effect varies between species, being positive, negative, or neutral.

### 4.3 Grouping of Species by Sensitivity to Light Pollution

Our studies have shown that photosynthetic traits (fluorescence yield based on fluorescence induction (Y(II)), photochemical quenching (qp) and electron transport efficiency (ETR) combined, and net photosynthetic yield based on carbon fixation) are the primary traits suitable for investigating the effects of light pollution in deciduous woody plants. Different species respond differently to excess illumination (Giavi et al. 2020; Zaimenko et al. 2023). The 19 tree species studied can be classified into four groups based on their sensitivity to artificial light at night: strongly positive response, positive response, neutrally affected, and negatively affected. Common linden, Japanese cherry, flowering ash and common hackberry are neutrally affected by 2.9 photosynthetically active radiation at 3000 K compared to 0.04 PAR. The empress tree, Japanese pagoda tree, elder, common alder and small‐leaved linden increase photosynthetic performance, while silver birch, sessile oak, tree‐of‐heaven, purple‐leaf plum tree and field maple have significantly higher night‐lighting capacity. The photosynthetic characteristics of the American plane tree are negatively affected by street light illumination. In their case, excess lighting at night is indeed light pollution. Trees grouped by Cathey and Campbell (1975) and Hightshoe (1988) according to light sensitivity and the present study are identical in nine species, of which four species show similarity: black locust as a species with high sensitivity to light pollution, small‐leaved linden and Japanese pagoda tree as a species with medium sensitivity to light pollution, and sessile oak as a species with low sensitivity to light pollution. The classification of the other five species (judas tree, common linden, common alder, silver birch and field maple) is opposite in the present study, but it should be noted that the previous groupings are based on the light conditions of the place of origin and differences in phenology depending on the street lamp types different from those of our study. The irradiance of the place of origin does not show any correlation with parameters affecting photosynthesis in plants. This refutes the claim by Chaney (2002) and Briggs (2006) that long‐day plants benefit from the additional light at night, while short‐day plants benefit from it as a negative environmental factor. Artificial light at night also leads to different responses in related species.

It should be noted that not all species in our studies have sufficient numbers of individuals to clearly detect a response to light pollution, but similar responses were observed for high numbers of individuals. In order to prove this, further individuals of species with low numbers need to be included in future studies. Furthermore, it is worthwhile to include additional species in the studies, as it can be seen that the responses to night‐time illumination are different, which would provide a more complex insight into the relationship between plant physiology and artificial light at night.

## 5 Conclusion

Our results show that deciduous trees in the town centre have different light sensitivity. These light sensitivities should be taken into account in landscaping and tree planting programmes, as the night‐time illumination of the urban environment is increasing. The lower photosynthetic efficiency of the illuminated parts of poorly selected species affects the amount of both primary and secondary metabolites, which can affect feeding interactions. Furthermore, a reduction in photosynthetic efficiency due to artificial light can also have a negative effect on the plant's defenses, which can reduce its resistance to pests. The latter two factors can lead to changes in urban ecosystems.

## Conflicts of Interest

The authors declare no conflicts of interest.

## Data Availability

The datasets of the current study are available in repository: https://osf.io/2rujy/.
